# Supplementary material for: Grafting of Thiazole Derivative on Chitosan Magnetite Nanoparticles for Cadmium Removal—Application for Groundwater Treatment
Source: Polymers (Basel). 2022 Mar 18;14(6):1240. doi: 10.3390/polym14061240 (PMC8954473; doi:10.3390/polym14061240)
Supplement: Supplementary file 1 [file polymers-14-01240-s001.zip › polymers-1610374-supplementary.pdf]

polymers

MDPI

Supplementary Materials

Grafting of Thiazole Derivative on Chitosan Magnetite Nanoparticles for Cadmium Removal—Application for Groundwater Treatment

Mohammed F. Hamza [1,2,*], Adel A.-H. Abdel-Rahman [3], Alyaa S. Negm [3], Doaa M. Hamad [3], Mahmoud S. Khalafalla [2], Amr Fouda [4], Yuezhou Wei [1,5,*], Hamada H. Amer [6], Saad H. Alotaibi [6] and Adel E.-S. Goda [7]

1 School of Nuclear Science and Technology, University of South China, Hengyang 421001, China.
2 Semi Pilot Plant Department, Nuclear Materials Authority, POB 530, El-Maadi, Cairo, Egypt; mahmoud-sayed24@yahoo.com
3 Chemistry Department, Faculty of Science, Menofia University, Shebin El-Kom 32511, Egypt; adelnassar63@yahoo.com (A.A.-H.A.-R.); meenmido000@gmail.com (A.S.N.); dodyhamad95@gmail.com (D.M.H.)
4 Botany and Microbiology Department, Faculty of Science, Al-Azhar University, Nasr City, Cairo 11884, Egypt; amr_fh83@azhar.edu.eg
5 School of Nuclear Science and Engineering, Shanghai Jiao Tong University, Shanghai, 200240, China;
6 Department of Chemistry, Turabah University College, Turabah, Taif University, P.O. Box 11099, Taif 21944, Saudi Arabia; h.amer@tu.edu.sa (H.H.A.); s.alosaimi@tu.edu.sa (S.H.A.)
7 Tanta Higher Institute of Engineering and Technology, Tanta 31739, Egypt; adelgoda1969@gmail.com (A.E.-S.G.)
* Correspondence: m_fouda21@hotmail.com (M.F.H.); yzwei@sjtu.edu.cn (Y.W.); Tel.: +2-01116681228 (M.F.H.); +86-771-322-4990 (Y.W.)

Citation: Hamza, M.F.; Abdel-Rahman,A,A,-H; Negm, A.S.; Hamad, D.M.; Khalafalla, M.S.; Fouda, A.; Wei, Y.; Amer, H.H.; Alotaibi, S.H.; Goda, A.E.-S. Grafting of Thiazole Derivative on Chitosan Magnetite Nanoparticles for Cadmium Removal—Application for Groundwater Treatment. *Polymers* **2022**, *14*, x. https://doi.org/10.3390/xxxxx

Academic Editor(s): Gregorio Cadenas-Pliego

Received: 8 February 2022
Accepted: 16 March 2022
Published: date

Publisher's Note: MDPI stays neutral with regard to jurisdictional claims in published maps and institutional affiliations.

Copyright: © 2022 by the authors. Submitted for possible open access publication under the terms and conditions of the Creative Commons Attribution (CC BY) license (https://creativecommons.org/licenses/by/4.0/).

Table S1. a. Reminder on equations used for modeling uptake kinetics [1,2] [3].

Model	Equation	Parameters	Ref.
PFORE	$q(t) = q_{eq,1}(1 - e^{-k_1 t})$	$q_{eq,1}$ (mmol g^{-1}): sorption capacity at equilibrium<br>k_1 (min^{-1}): apparent rate constant of PFORE	[3]
PSORE	$q(t) = \frac{q_{eq,2}^2 k_2 t}{1 + k_2 q_{eq,2} t}$	$q_{eq,2}$ (mmol g^{-1}): sorption capacity at equilibrium<br>k_2 (g mmol^{-1} min^{-1}): apparent rate constant of PSORE	[3]
RIDE	$\frac{q(t)}{q_{eq}} = 1 - \sum_{n=1}^{\infty} \frac{6\alpha(\alpha+1)\exp\left(\frac{-D_e q_n^2}{r^2} t\right)}{9 + 9\alpha + q_n^2 \alpha^2}$<br>With q_n being the non-zero roots of<br>$\tan q_n = \frac{3\, q_n}{3 + \alpha q_n^2}$ and $\frac{m\, q}{V\, C_0} = \frac{1}{1 + \alpha}$	D_e (m^2 min^{-1}) : Effective diffusivity coefficient	[1]

(m (g): mass of sorbent; V (L): volume of solution; C_0 (mmol L^{-1}): initial concentration of the solution).

Table S1. b. Reminder on equations used for modeling sorption isotherms [2, 4, 5].

Model	Equation	Parameters	Ref.
Langmuir	$q_{eq} = \frac{q_{m,L} C_{eq}}{1 + b_L C_{eq}}$	$q_{m,L}$ (mmol g^{-1}): Sorption capacity at saturation of monolayer<br>b_L (L mmol^{-1}): Affinity coefficient	[2]
Freundlich	$q_{eq} = k_F C_{eq}^{1/n_F}$	k_F and n_F: empirical parameters of Freundlich equation	[2]
Sips	$q_{eq} = \frac{q_{m,S} b_S C_{eq}^{1/n_S}}{1 + b_S C_{eq}^{1/n_S}}$	$q_{m,L}$, b_S and n_S: empirical parameters of Sips equation (based on Langmuir and Freundlich equations)	[2]
Temkin	$q_{eq} = \frac{R\,T}{b_T} \ln(A_T C_{eq})$	b_T: J kg mol^{-2}, Temkin isotherm constant<br>A_T: L mol^{-1}, Temkin isotherm equilibrium constant	[5]

Akaike Information Criterion, AIC [6]:

$$AIC = N \ln\left(\frac{\sum_{i=0}^{N}\left(y_{i,exp.} - y_{i,model}\right)^2}{N}\right) + 2N_p + \frac{2N_p(N_p + 1)}{N - N_p - 1}$$

Where N is the number of experimental points, N_P the number of model parameters, $y_{i,exp.}$ and $y_{i,model}$ the experimental and calculated values of the tested variable.

Table S2. Assignments of the metal concentration comparing with the MCL values for drinking water and livestock drinking water.

	Initial conc.	MCL (D.W)	MCL (L.W)	D.W increased time	L.W increased time
Cd (II)	1.97	0.003	0.05	656.67	39.4
Zn (II)	7.64	5	24	1.528	0.318
pb (II)	0.997	0.01	0.1	99.7	9.97
Hg(II)	0.759	0.002	0.01	379.5	75.9
Cu(II)	2.19	1.3	0.5	1.685	4.38
Al(III)	6.943	0.2	5	34.715	1.389

Table S3. Textural properties of MCH and MCH-ATA.

Parameter	MCH	MCH-ATA
S_{BET} (m^2 g^{-1})	21.17	22.5
V_P (cm^3 STP g^{-1})	7.14	7.7

Table S4. Elemental analysis of MCH and MCH-ATA sorbents.

	MCH					MCH-ATA					
	C	N	H	O	Fe	C	N	H	O	Fe	S
[%]	26.97	4.28	4.96	31.61	32.18	18.5	6.09	4.18	35.94	30.96	4.33
Mmol	22.456	3.056	49.206	19.758	5.762	15.404	4.348	41.468	22.464	5.544	1.35

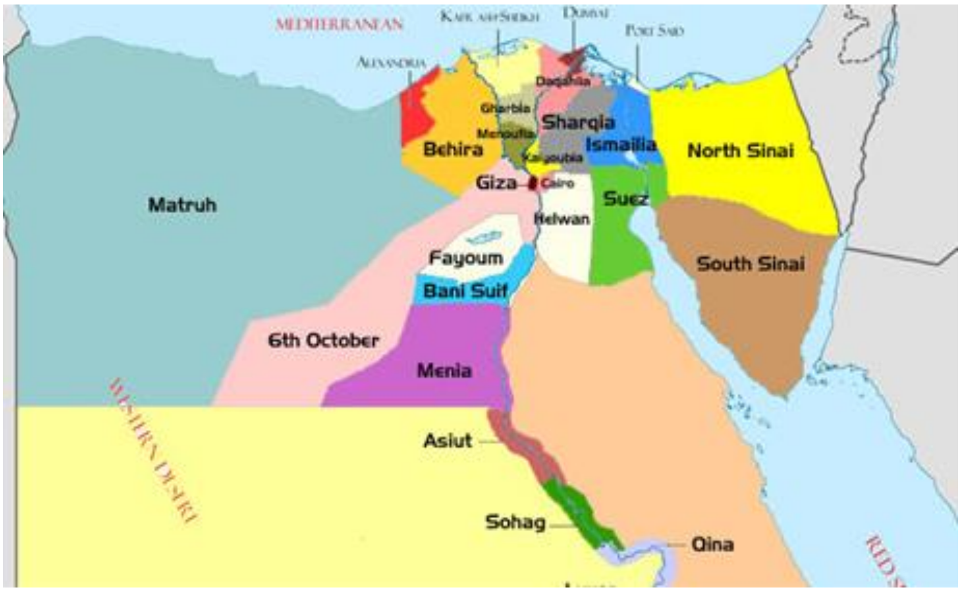

Figure S1. Tora Hellwan area where real contaminated samples were collected.

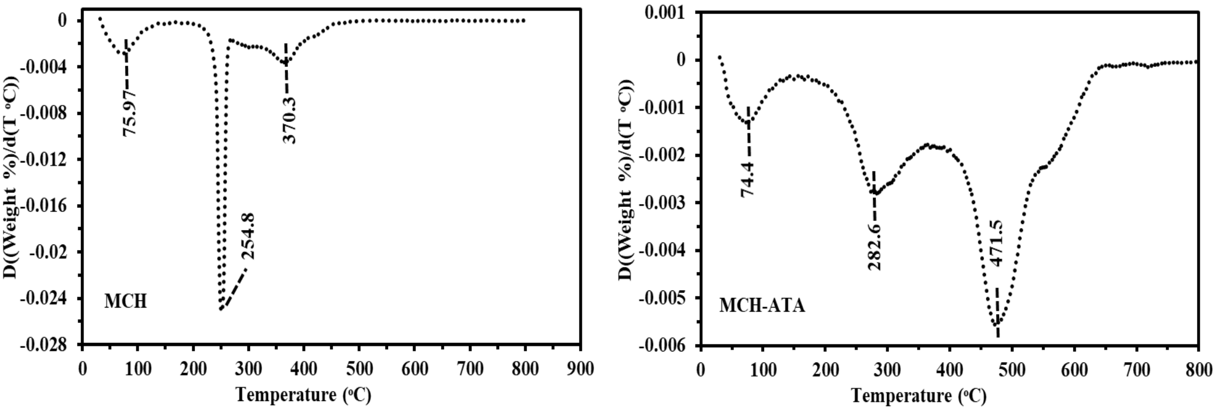

Figure S2. DrTG data for MCH and after functionalization (MCH-ATA).

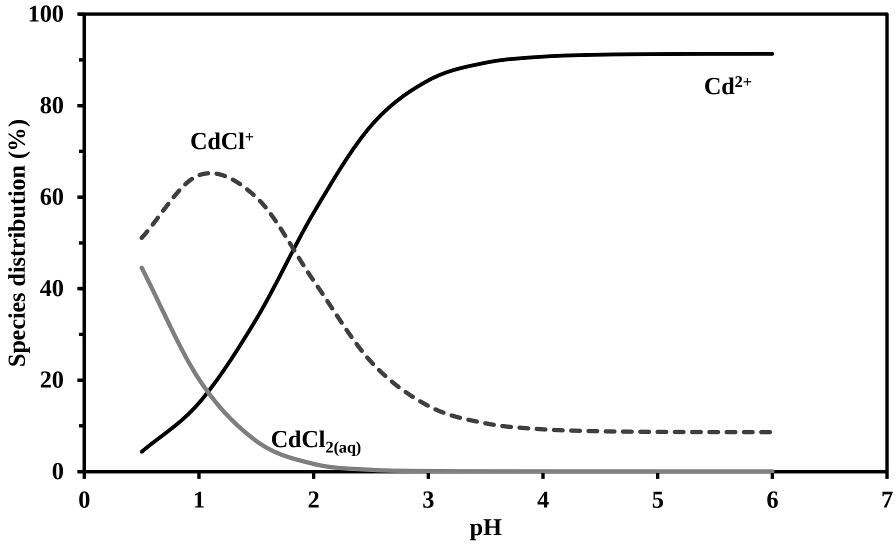

Figure S3. Speciation diagrams for Cd(II) under the experimental conditions that selected for the study of pH effect (calculation using Visual Minteq, [7]).

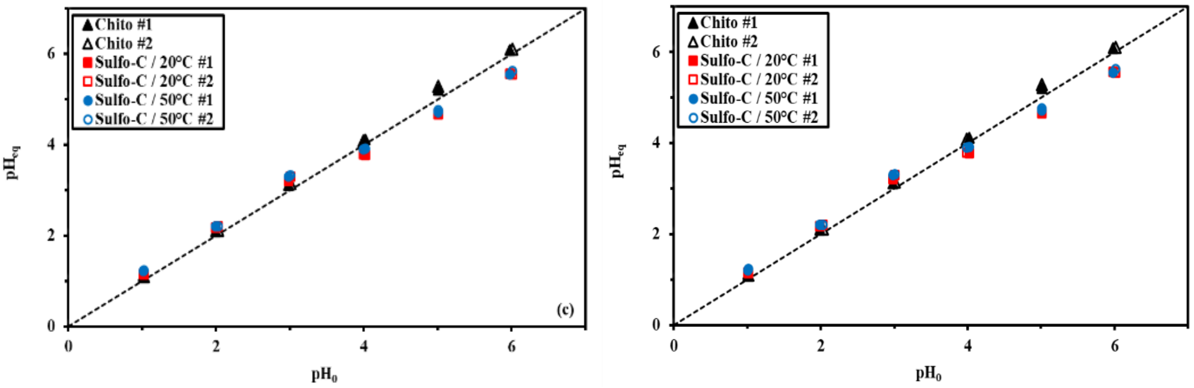

Figure S4. Variation in the pH (from 1-5) for MCH and MCH-ATA.

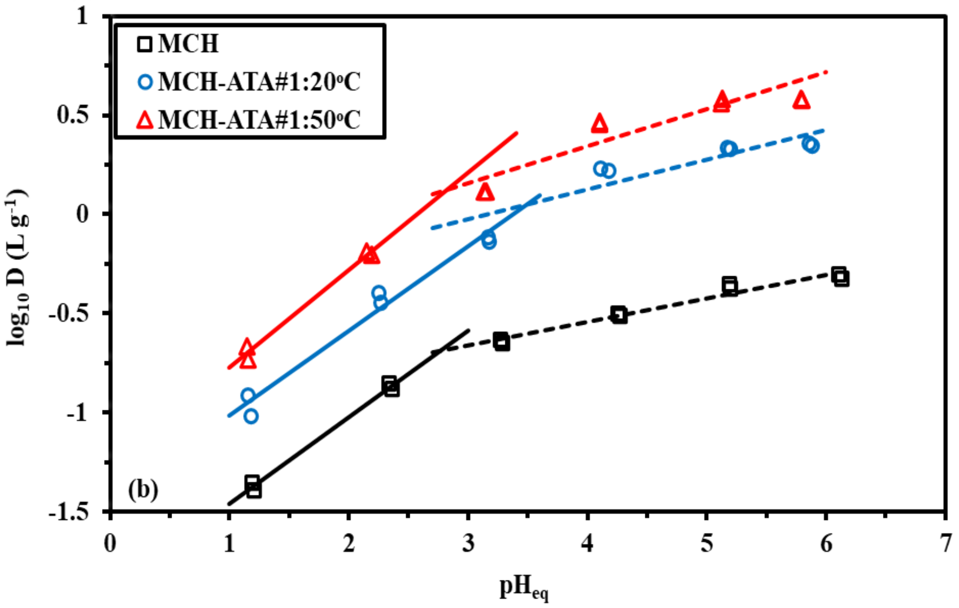

Figure S5. Plotting of pH_{eq} *vs* Log10D at 20 and 50°C.

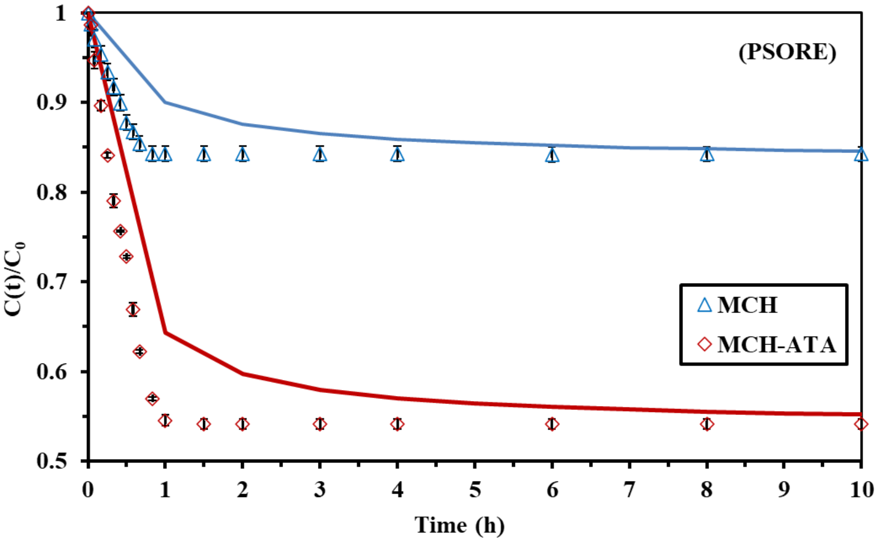

Figure S6. Average values of kinetic modeling with error bars of the triplicated experimentsfor PSORE of MCH (a) and MCH-ATA(b).

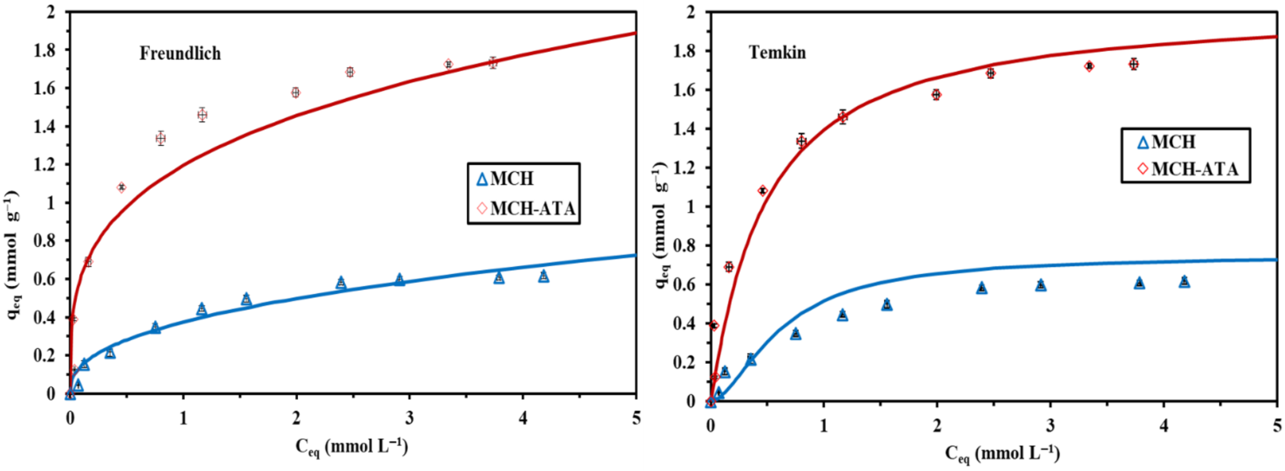

Figure S7. Average values of Freundlich and Temkin equations with error bars of the triplicated experiments for Cd(II) sorption on MCH and MCH-ATA sorbents.

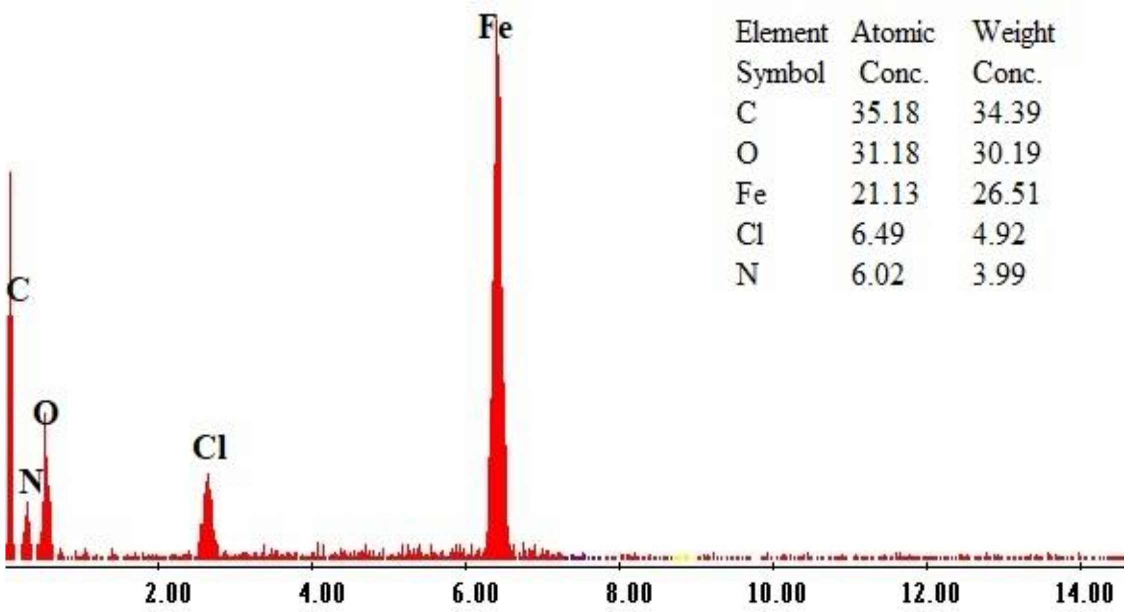

Figure S8. EDX analysis of the MCH-ATA sorbent after 5 cycles of sorption desorption.

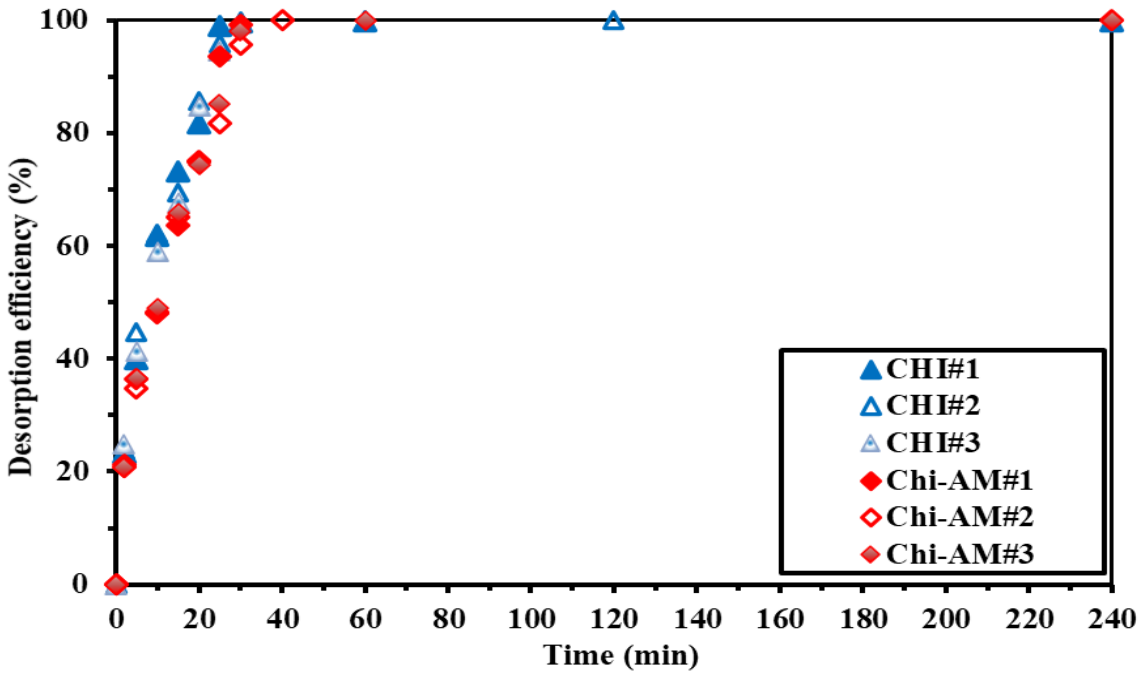

Figure S9. Desorption kinetics of MCH and MCH-ATA using 0.2MHCl solution.

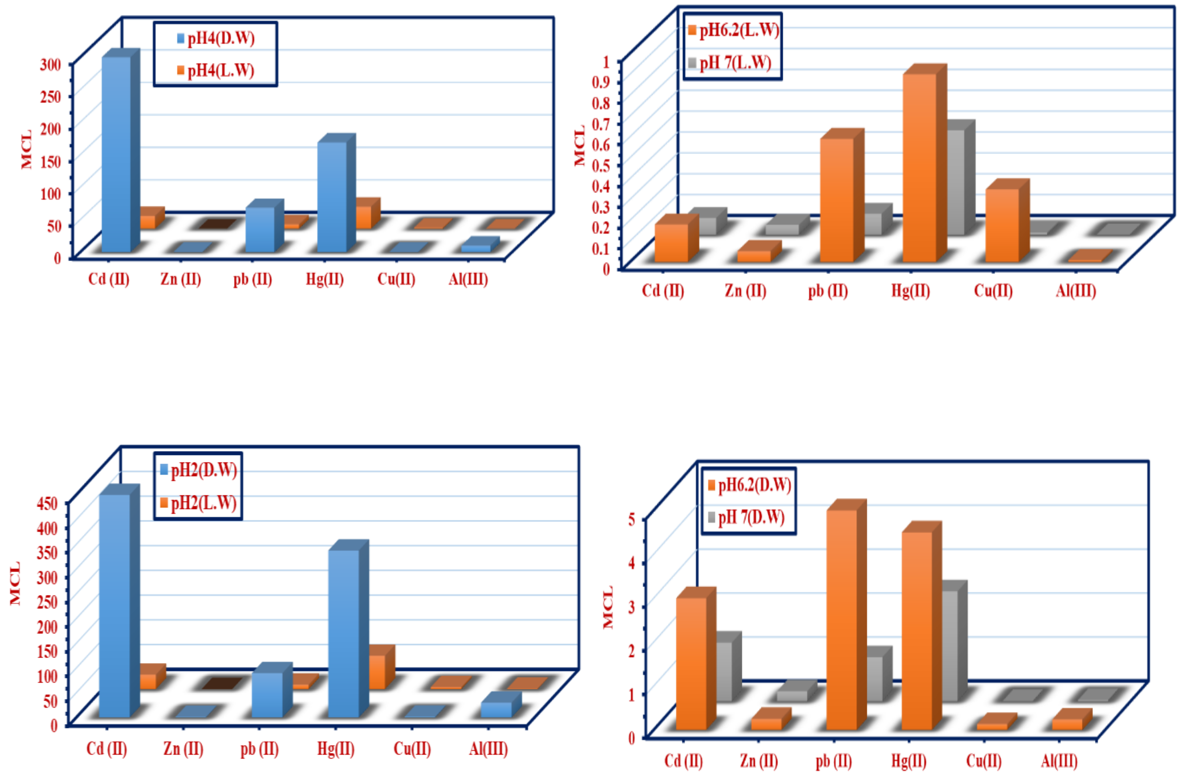

Figure S10. Comparison of the MCL levels for drinking water and Livestock drinking water for MCH-ATA at different pH values.

References

1. Crank, J. *The Mathematics of Diffusion*, 2nd. ed.; Oxford University Press: Oxford, U.K., 1975; p. 414.
2. Tien, C. *Adsorption Calculations and Modeling*; Butterworth-Heinemann: Newton, MA, 1994; p. 243.
3. Ho, Y.S.; McKay, G. Pseudo-second order model for sorption processes. *Process Biochem.* **1999**, *34*, 451-465, doi:10.1016/S0032-9592(98)00112-5.
4. Lima, É.C.; Dehghani, M.H.; Guleria, A.; Sher, F.; Karri, R.R.; Dotto, G.L.; Tran, H.N. CHAPTER 3 - Adsorption: Fundamental aspects and applications of adsorption for effluent treatment. In *Green Technologies for the Defluoridation of Water*, Hadi Dehghani, M., Karri, R., Lima, E., Eds.; Elsevier: 2021; pp. 41-88.
5. Buema, G.; Lupu, N.; Chiriac, H.; Ciobanu, G.; Bucur, R.D.; Bucur, D.; Favier, L.; Harja, M. Performance assessment of five adsorbents based on fly ash for removal of cadmium ions. *J. Mol. Liq.* **2021**, *333*, 115932, doi:10.1016/j.molliq.2021.115932.
6. Falyouna, O.; Eljamal, O.; Maamoun, I.; Tahara, A.; Sugihara, Y. Magnetic zeolite synthesis for efficient removal of cesium in a lab-scale continuous treatment system. *J. Colloid Interface Sci.* **2020**, *571*, 66-79, doi:10.1016/j.jcis.2020.03.028.
7. Gustafsson, J.P. *Visual MINTEQ, ver. 3.1*, ver. 3.1; KTH, Royal Institute of Technology, (url: https://vminteq.lwr.kth.se/; accessed May 2017): Stockholm, Sweden, 2013.
